# Ultra‐High Temperature Calcination of Crystalline α‐Fe_2_O_3_ and Its Nonlinear Optical Properties for Ultrafast Photonics

**DOI:** 10.1002/advs.202500896

**Published:** 2025-03-17

**Authors:** Qingxi Zhao, Qingling Tang, Hongwei Chu, Zhongben Pan, Han Pan, Shengzhi Zhao, Dechun Li

**Affiliations:** ^1^ School of Information Science and Engineering and Key Laboratory of Laser and Infrared System of Ministry of Education Shandong University Qingdao 266237 China

**Keywords:** mode‐locking, nonlinear optical properties, phase transition, ultra‐high temperature, α‐Fe_2_O_3_

## Abstract

As a typical transition metal oxide, α‐Fe_2_O_3_ has garnered significant attention due to its advantages in nonlinear optical applications, such as strong third‐order nonlinearity and fast carrier recovery time. To delve into the nonlinear optical properties of α‐Fe_2_O_3_, crystalline α‐Fe_2_O_3_ materials with different microstructures are prepared. The nonlinear optical features of α‐Fe_2_O_3_ calcined at the previously unexplored ultra‐high temperature of >1100°C are emphasized. It is found that α‐Fe_2_O_3_ exposed to ultra‐high temperatures undergoes the phase transition, leading to the formation of Fe_3_O_4_. Subsequently, the nonlinear absorption coefficient is measured as −0.6280 cm GW^−1^ at 1.5 µm. The modulation depth and saturation intensity for the Fe_2_O_3_‐based saturable absorber at 1.5 µm are 4.20% and 13.94 MW cm^−2^, respectively. Ultimately, the incorporation of the Fe_2_O_3_‐based saturable absorber into an Er‐doped fiber laser cavity resulted in the achievement of both conventional soliton mode‐locking operation with a central wavelength of 1560.3 nm and a pulse duration of 1.13 ps, as well as the dissipative soliton resonance mode‐locking operation with a central wavelength near 1564.0 nm. Overall, the phase transition and the nonlinear optical features in iron oxides under ultra‐high temperatures are revealed, indicating the great potential in advanced ultrafast photonic applications.

## Graphical abstract

This study focuses on exploring the nonlinear optical properties of α‐Fe_2_O_3_ material calcined at ultra‐high temperatures. It is discovered that the α‐Fe_2_O_3_ material underwent the phase transition at ultra‐high temperatures. Then, the α‐Fe_2_O_3_ material calcined at 1100 °C is successfully integrated as a saturable absorber into an erbium‐doped fiber laser, achieving conventional soliton mode‐locking operation and dissipative soliton resonance mode‐locking operation within the C‐band.

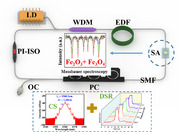

## 1 Introduction

Nonlinear optical (NLO) materials are crucial components in various optoelectronic devices, with their performance often dictating the overall efficacy of these devices. In recent years, transition metal oxides (TMOs) have emerged as premier candidates for nonlinear optoelectronics due to their advantageous properties, including strong third‐order nonlinearity, ultrafast carrier recovery time, broad absorption bandwidth, strong light‐matter interaction, and elevated exciton binding energy.^[^ ^1^, ^2^, ^3^, ^4^, ^5^, ^6^, ^7^, ^8^, ^9^, ^10^, ^11^, ^12^, ^13^, ^14^ ^]^ Among TMOs, Fe_2_O_3_ has garnered significant attention owing to its natural abundance and exceptional thermochemical stability. There exist four crystalline forms of Fe_2_O_3_: α‐Fe_2_O_3_, β‐Fe_2_O_3_, γ‐Fe_2_O_3_, and ε‐Fe_2_O_3_.^[^ ^15^ ^]^ Notably, hematite (α‐Fe_2_O_3_) is an *n*‐type semiconductor characterized by a bandgap of 2.1 eV and a rhombohedral corundum structure, recognized as the most stable form of iron oxide.^[^ ^16^, ^17^ ^]^ It exhibits complex magnetic behavior that includes antiferromagnetism alongside weak ferromagnetism. Furthermore, due to its low cost and environmental friendliness, it has been widely applied in catalysis,^[^ ^18^, ^19^ ^]^ water treatment,^[^ ^20^ ^]^ electrochemistry,^[^ ^21^, ^22^ ^]^ water splitting,^[^ ^23^, ^24^ ^]^ nonlinear optics,^[^ ^25^, ^26^, ^27^ ^]^ and other fields. Thanks to the wide application of α‐Fe_2_O_3_ material, to date, numerous synthesis methods have been reported for producing α‐Fe_2_O_3_ material, including sol–gel method,^[^ ^28^ ^]^ hydrothermal synthesis method,^[^ ^29^ ^]^ solvothermal method,^[^ ^30^ ^]^ in situ synthesis method,^[^ ^31^ ^]^ template‐based method,^[^ ^32^ ^]^ and electrochemical anodization method.^[^ ^33^ ^]^

Generally speaking, α‐Fe_2_O_3_ is frequently prone to lose surface oxygen atoms when undergoing heat treatment or chemical reactions during the preparation process, forming oxygen vacancy (O_V_) defects.^[^ ^34^, ^35^ ^]^ These oxygen vacancy defects will cause the Fe/O ratio of α‐Fe_2_O_3_ to increase, enabling α‐Fe_2_O_3_ material to have more surface active sites and ultimately enhance its performance in catalysis, electrochemistry, and ultrafast photonics.^[^ ^36^, ^37^, ^38^, ^39^, ^40^, ^41^ ^]^ Therefore, it is of great significance to regulate the concentration of O_V_ in α‐Fe_2_O_3_ material. To achieve this goal, people usually adjust the oxygen vacancy concentration and Fe/O ratio in α‐Fe_2_O_3_ material by controlling certain parameters during the preparation process, such as the preparation temperature and holding time. Nevertheless, the aforementioned methods for preparing α‐Fe_2_O_3_ material often have drawbacks like high‐cost, low yield, complex procedures, and poor controllability of oxygen vacancy concentration when preparing the α‐Fe_2_O_3_ material with adjustable oxygen vacancy concentration, which restricts the large‐scale production and application of modified α‐Fe_2_O_3_ material. As we all know, the high‐temperature calcination method is a simple and low‐cost process to prepare oxygen vacancy defects. During the high‐temperature calcination, the lattice oxygen on the material's surface is prone to loss, thereby forming oxygen vacancy defects.^[^ ^34^, ^42^, ^43^ ^]^ It is easy to regulate the concentration of oxygen vacancy and the Fe/O ratio in the α‐Fe_2_O_3_ material by controlling the calcination temperature and duration of the calcination process of α‐Fe_2_O_3_ material. Additionally, by adjusting the calcination temperature and duration, the crystallinity of α‐Fe_2_O_3_ material can also be regulated. Furthermore, due to the existence of various iron oxides with different Fe/O ratios, high‐temperature calcination may also cause phase transition among iron oxides, which is also beneficial for the improvement of ultrafast nonlinear optical properties.

Herein, FeOOH material was employed as the precursor, and a series of crystalline α‐Fe_2_O_3_ materials with diverse microstructures were fabricated via the high‐temperature calcination method at different calcination temperatures. In addition, these α‐Fe_2_O_3_ materials were characterized and tested for nonlinear optical properties. Based on the characterization results, it was discovered that when the calcination temperature exceeds 1000 °C, the calcination products encompass not only α‐Fe_2_O_3_ but also a portion of Fe_3_O_4_. Based on the results of nonlinear optical properties testing, we chose to focus on the calcined product with the calcination temperature of 1100 °C (named Fe_2_O_3_‐H). The nonlinear absorption coefficient is −0.628 cm GW^−1^ by utilizing a home‐built Z‐scan setup in the waveband of 1.5 µm. The modulation depth and saturation intensity of the Fe_2_O_3_‐H saturable absorber (SA) at 1.5 µm were 4.20% and 13.94 MW cm^−2^ with a home‐built I‐scan setup. Finally, the Fe_2_O_3_‐H SA was incorporated into the Er‐doped fiber laser cavity to achieve the conventional soliton mode‐locking operation with a central wavelength of 1560.3 nm and a pulse duration of 1.13 ps, and the dissipative soliton resonance mode‐locking operation with a central wavelength near 1564.0 nm, respectively. The experimental results demonstrate that α‐Fe_2_O_3_ materials calcined at ultra‐high temperatures possess excellent nonlinear optical properties and great application potential in ultrafast photonics. This work paves the way for the investigation of the phase transition of iron oxides at ultra‐high temperatures and their applications in the field of nonlinear optics, which will be beneficial for the future application of iron oxides in advanced ultrafast photonics and optoelectronics.

## 2 Results and Discussion

### 2.1 DFT Simulation

**Figure 1:**
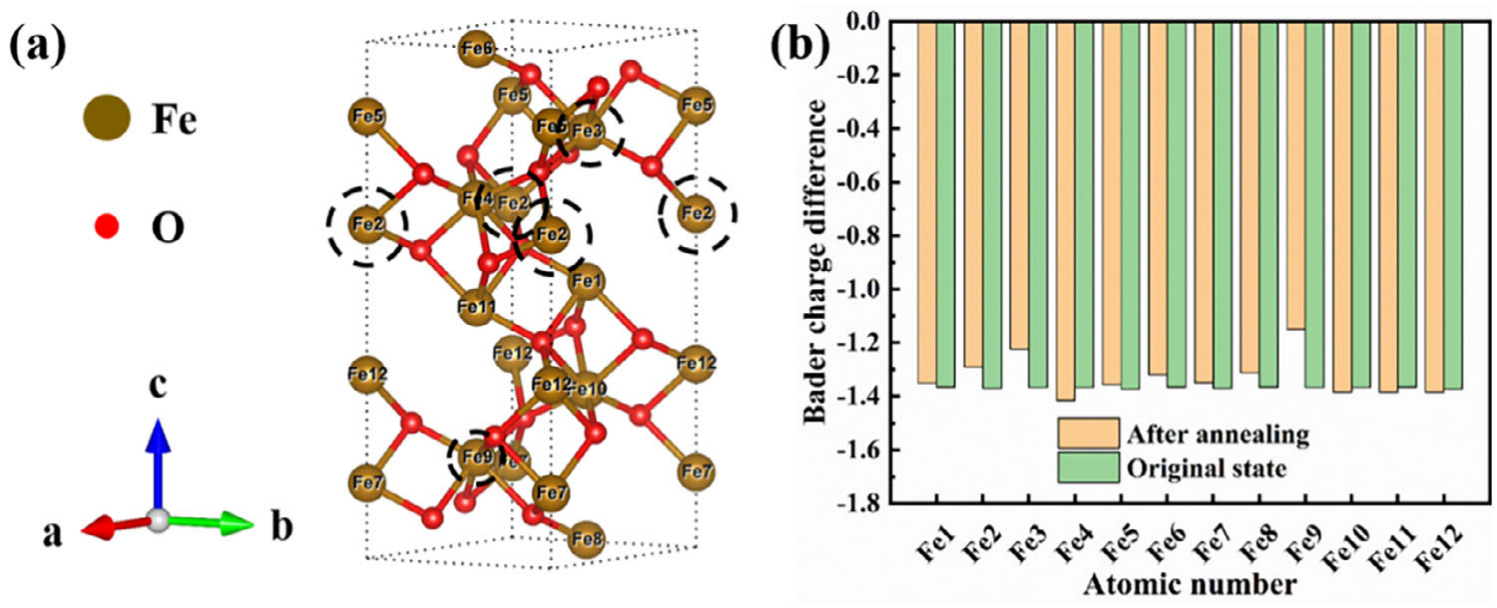
a) The cell diagram of α‐Fe_2_O_3_ material annealed at ultra‐high temperature; b) The bader charge difference between α‐Fe_2_O_3_ at room temperature and annealed at ultra‐high temperature.

In order to better understand the influence of ultra‐high temperature calcination on the electronic properties and phase transitions of α‐Fe_2_O_3_ materials, we used VASP to perform first‐principle calculations and study the difference between the electron gain and loss of α‐Fe_2_O_3_ at ultra‐high temperature and at room temperature, as shown in **Figure** **1**. In Figure 1a, we show the cell diagram of α‐Fe_2_O_3_ material after ultra‐high temperature annealing. In Figure 1b, we observe that some Fe atoms in a cell of α‐Fe_2_O_3_ material undergo different electron transfers after ultra‐high temperature calcination. This result suggests that a part of α‐Fe_2_O_3_ may undergo the phase transition at ultra‐high temperatures, leading to the formation of different iron oxide phases. In addition, the observed electron transfer may have an important effect on the nonlinear optical properties of the α‐Fe_2_O_3_ material, which needs to be verified experimentally.

### 2.2 Characterization of α‐Fe_2_O_3_ Materials

**Figure 2:**
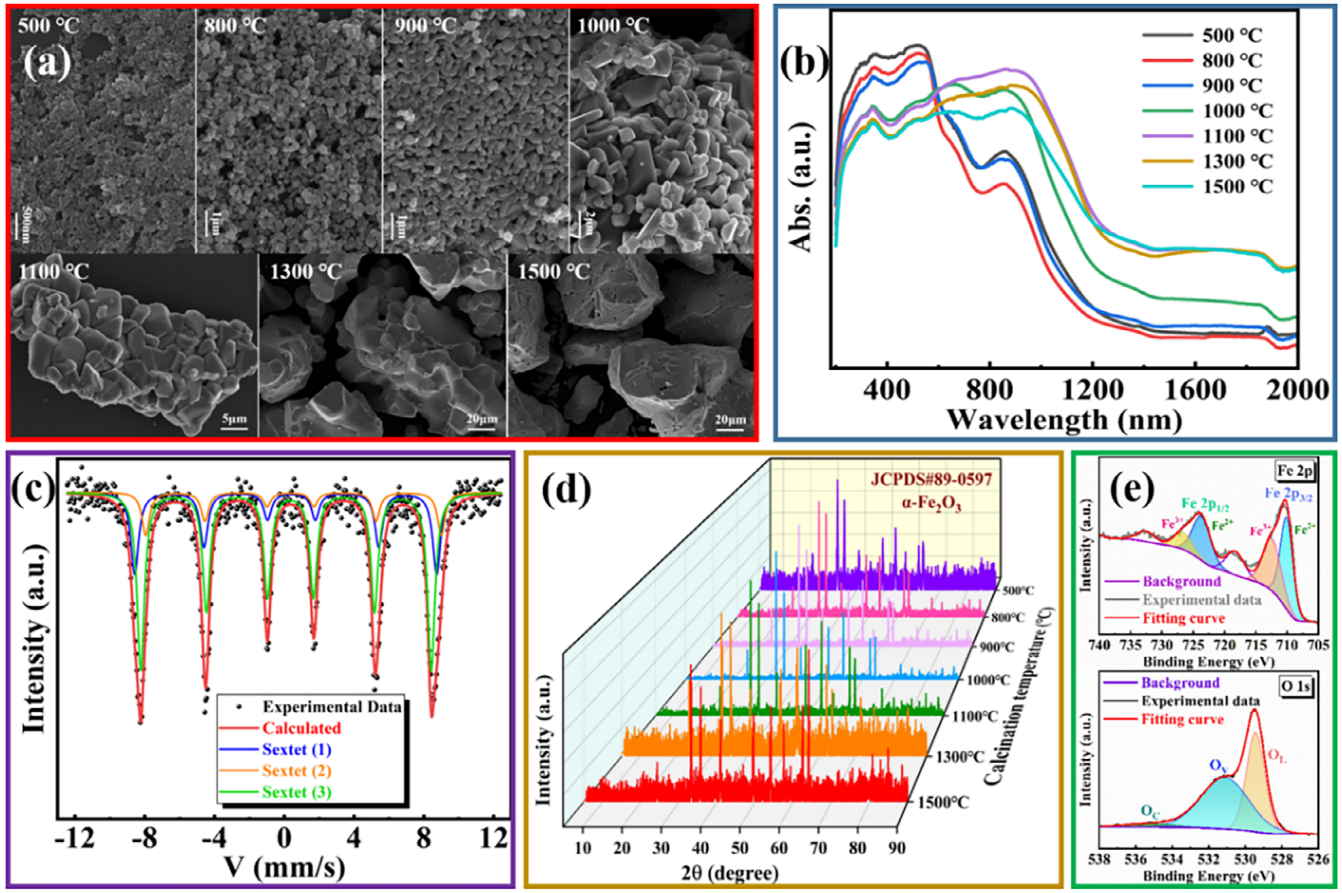
a) SEM images of calcined α‐Fe_2_O_3_ materials at different calcination temperatures; b) UV–vis–IR absorption spectra of different α‐Fe_2_O_3_ materials; c) Mossbauer spectroscopy of Fe_2_O_3_‐H material; d) XRD patterns of different α‐Fe_2_O_3_ materials; e) XPS spectra of Fe_2_O_3_‐H material.

A series of crystalline α‐Fe_2_O_3_ materials with different calcination temperatures were prepared by the high‐temperature calcination method. The specific preparation process is shown in “Experimental Section.” During the preparation of α‐Fe_2_O_3_ materials through high‐temperature calcination, the FeOOH precursor was annealed within a range of temperatures, and a series of calcined products with diverse microstructures were obtained. It is notable that when the calcination temperature exceeds 1000 °C, the calcined products demonstrate entirely different characteristics: First, the color of the calcined products is no longer the reddish‐brown exhibited by other products, but rather shows a dark blue performance. Meanwhile, they also exhibit strong magnetism not possessed by other calcined products, which has not been reported in previous studies. The characterization results related to the α‐Fe_2_O_3_ materials are shown in **Figure** **2** and Supporting Information.

The scanning electron microscope (SEM) images of the α‐Fe_2_O_3_ materials are shown in Figure 2a. It can be noticed that the appearance and size of the obtained α‐Fe_2_O_3_ materials also vary with the alteration of the calcination temperature. When the calcination temperature is 500 °C, the calcined α‐Fe_2_O_3_ material manifests an irregular porous flocculation structure. The formation of the porous structure can be accounted for as follows: During the high‐temperature calcination process, the removal of hydroxyl groups and water causes the collapse and reorganization of the FeOOH structure, ultimately resulting in the emergence of the porous structure in the α‐Fe_2_O_3_ material.^[^ ^41^, ^42^, ^43^, ^44^, ^45^, ^46^, ^47^ ^]^ When the calcination temperature is 800 °C, the obtained α‐Fe_2_O_3_ material appears as irregularly dispersed particles with a particle size of ≈300 nm. With the increase of the calcination temperature, the particle size of the calcined products will continue to grow, which can be attributed to the growth, aggregation, and fusion of the dispersed particles after calcination at a higher temperature,^[^ ^46^, ^48^ ^]^ as well as demonstrated by the calcined products obtained at 1000 °C. When the calcination temperature is greater than 1000 °C, the aggregation and fusion of particles in the calcination products become more pronounced, leading to the continuous increase of the single particle volume and the continuous decrease of the single particle surface area. Figure S1 (Supporting Information) displays the microstructure images of Fe_2_O_3_‐H material obtained at an ultra‐high calcination temperature of 1100 °C. The SEM images of the Fe_2_O_3_‐H material are depicted in Figure S1a,c (Supporting Information). It can be observed that the Fe_2_O_3_‐H material exhibits an uneven block structure, with a micron‐sized scale and mutual aggregation among the blocks. The transmission electron microscope (TEM) images of Fe_2_O_3_‐H material are shown in Figure S1b,d (Supporting Information). In Figure S1b (Supporting Information), the homogeneous internal structure of the irregular block can be seen, while in the high‐resolution transmission electron microscopy (HRTEM) image shown in Figure S1d (Supporting Information), a lattice spacing of 2.75 Å, corresponding to the (104) lattice plane of α‐Fe_2_O_3_ material, can be observed, illustrating the good crystallism of the Fe_2_O_3_‐H material.

Figure 2b presents the absorption spectra of α‐Fe_2_O_3_ materials within the range of 200–2000 nm. It can be observed that all of the α‐Fe_2_O_3_ materials possess broadband absorption characteristics in the near‐infrared wavelength range. Nevertheless, the absorption of α‐Fe_2_O_3_ materials obtained through calcination at ultra‐high temperatures is significantly stronger than that of other α‐Fe_2_O_3_ materials in the near‐infrared band, suggesting the positive modification effect of ultra‐high temperature calcination on α‐Fe_2_O_3_ materials in the field of nonlinear optics. Additionally, it can also be noted that the absorption spectra of α‐Fe_2_O_3_ materials resulting from ultra‐high temperature calcination are similar, and the absorption spectra of α‐Fe_2_O_3_ materials with calcination temperatures less than 1000 °C are also mutually similar. Concurrently, the α‐Fe_2_O_3_ materials obtained at the calcination temperature below 1000 °C appear reddish‐brown and have no strong magnetism, while the α‐Fe_2_O_3_ materials obtained through ultra‐high temperature calcination show dark blue and have strong magnetism. Combined with our calculation results, we hypothesize that apart from the production of α‐Fe_2_O_3_, other iron oxide materials may also emerge during the ultra‐high temperature calcination process, which has not been reported in previous studies. In Figure S2 (Supporting Information), the absorption spectrum of Fe_2_O_3_‐H material is provided, and the illustration is the macroscopic diagram of Fe_2_O_3_‐H material. Furthermore, the spectral line jitter near the wavelength of 400 nm is caused by the change of the lamp in the test instrument.

To explore the material composition of the α‐Fe_2_O_3_ materials obtained under ultra‐high temperature calcination, we conducted the Mossbauer spectroscopy for Fe_2_O_3_‐H material, and the result is presented in Figure 2c. Mossbauer spectroscopy is a frequently employed method for investigating the local magnetic behavior of Fe atoms in materials and their oxidation states. As can be seen from Figure 2c, the Fe_2_O_3_‐H material exhibits three sextet states, demonstrating the existence of other iron oxides apart from α‐Fe_2_O_3_ in the material. According to the test results of the Mossbauer spectroscopy (as shown in **Table** **1**), for the third sextet with the highest proportion, the value of its quadrupole splitting (QS, −0.26 mm s^−1^) indicates that the material possesses weak ferromagnetism. The value of its isomer shifts (IS, 0.343 mm s^−1^) and the value of its hyperfine field (B_hf_, 51.79 T) all correspond to the hematite phase,^[^ ^49^ ^]^ which is consistent with our previous analysis results. Additionally, for the remaining two sextet states, their corresponding IS values are 0.353 and 0.553 mm s^−1^ respectively, corresponding to Fe^3+^ ions and Fe^2+^ ions.^[^ ^50^, ^51^ ^]^ The ratio of the two is ≈2:1, which is in accordance with the ratio of Fe^3+^ ions to Fe^2+^ ions in Fe_3_O_4_. Simultaneously, since at ultra‐high temperatures, Fe_2_O_3_ may decompose to generate Fe_3_O_4_ and O_2_. Therefore, we conclude that the Fe_2_O_3_‐H material should contain Fe_3_O_4_ in addition to α‐Fe_2_O_3_. This also explains the reason why the Fe_2_O_3_ materials under ultra‐high temperature calcination change color and demonstrate strong magnetism.

**Table 1:** Mossbauer spectroscopy test results.

–	IS[mm/s]	QS[mm/s]	H[T]	Area[%]
Sextet(1)	0.353	−0.33	53.82	26.2
Sextet(2)	0.553	0.17	52.68	13.2
Sextet(3)	0.343	−0.26	51.79	60.6

In order to investigate the impact of calcination temperature on materials’ crystallinity, α‐Fe_2_O_3_ materials prepared were characterized by X‐ray diffraction (XRD), and the results are displayed in Figure 2d. It can be perceived that the diffraction peaks of all materials correspond to the standard diffraction pattern of α‐Fe_2_O_3_ (JCPDS#89‐0597). Meanwhile, when the temperature is lower than 1000 °C, the crystallinity of the materials improves with the increase of calcination temperature. This is also manifested by the fact that the peak at 2θ = 33.1° becomes sharper and more intense. All these phenomena suggest that α‐Fe_2_O_3_ crystal grains grow significantly with the increase of calcination temperature, leading to better crystallinity. Additionally, when the temperature is higher than 1000 °C, the crystallinity of the calcined products deteriorates. The possible causes are as follows: A part of α‐Fe_2_O_3_ materials decompose at ultra‐high temperatures, and the ultra‐high temperature environment will also damage the original crystal structure of the α‐Fe_2_O_3_ materials, resulting in poorer crystallinity. The XRD characterization result of Fe_2_O_3_‐H material was presented in Figure S3 (Supporting Information), and it can be observed that the Fe_2_O_3_‐H material is polycrystalline. The peak at 2θ = 33.1° corresponds to the (104) lattice plane of α‐Fe_2_O_3_, which is in accordance with our previous HRTEM result. It is worth noting that the presence of α‐Fe_2_O_3_ can only be determined through the XRD pattern of the Fe_2_O_3_‐H material. The reason for this phenomenon is elucidated as follows: During the materials preparation process at ultra‐high temperatures, Fe_3_O_4_ may form fewer crystals or exist as a short‐range ordered phase along the hematite crystals, thereby not presenting significant peaks in the XRD pattern.^[^ ^49^ ^]^

We also found that Fe_2_O_3_‐H material undergoes the phase transition at ultra‐high temperatures, and has the best crystallinity after calcination at ultra‐high temperatures. In order to further investigate the Fe_2_O_3_‐H material, X‐ray photoelectron spectroscopy (XPS) was employed and the results are presented in Figure 2e. It can be observed that the Fe 2p energy spectrum predominantly exhibits two asymmetric peaks, corresponding respectively to the Fe 2p_1/2_ peak and Fe 2p_3/2_ peak, with their binding energies concentrating at ≈724 and 710 eV. Each asymmetric peak can be decomposed into two fitting peaks, among which the one with the higher binding energy pertains to Fe^3+^ ions, and the one with the lower binding energy pertains to Fe^2+^ ions.^[^ ^52^, ^53^ ^]^ Additionally, the two peaks of binding energy concentrating at ≈732 and 719 eV respectively correspond to the satellite peaks of Fe 2p_1/2_ peak and Fe 2p_3/2_ peak.^[^ ^54^ ^]^ As the Fe_2_O_3_‐H material is fabricated by the high‐temperature calcination method, and during the material calcination process, α‐Fe_2_O_3_ material readily loses the oxygen atoms on the surface to form oxygen vacancy, resulting in a certain amount of oxygen vacancy defects on the surface of the Fe_2_O_3_‐H material. The formation of oxygen vacancy on the surface will also induce the formation of Fe^2+^ ions on the surface, as depicted in the following equation: 2Fe^3+^ → 2Fe^2+^ + O_V_. This leads to a significant proportion of Fe^2+^ ions in the XPS analysis results used to detect the valence states of elements on the surface of Fe_2_O_3_‐H material. In addition, the O 1s energy spectrum can be decomposed into three principal peaks, where the peak at ≈529.2 eV is associated with lattice oxygen (O_L_), the peak at ≈531.5 eV corresponds to oxygen vacancy, and the peak at ≈535.0 eV can be attributed to chemisorbed oxygen (O_C_).^[^ ^55^, ^56^ ^]^ To verify the influence of calcination temperature on material composition and elemental valence states, XPS tests were carried out on all calcined products, and the fitting results were summarized in Table S1 (Supporting Information). By analyzing the data, it is found that the content of Fe^2+^ ions decreases and the content of O_V_ decreases with the increase of calcination temperature below 1000 °C. In combination with the SEM images shown in Figure 2a, the reason might be that with the increase of calcination temperature, the particle size of the materials becomes larger and the surface area becomes smaller, thereby the oxygen vacancy formed by the loss of oxygen atoms from the surface becomes less. When the calcination temperature is greater than 1000 °C, the O_V_ content of the material remains essentially unchanged, while the Fe^2+^ ions content increases with the increase of the calcination temperature, indicating that the reaction of Fe_3_O_4_ formation becomes more intense with the increase of temperature under an ultra‐high temperature environment.

### 2.3 Nonlinear Optical Response

#### 2.3.1 In‐Line Balanced Twin‐Detector System

**Figure 3:**
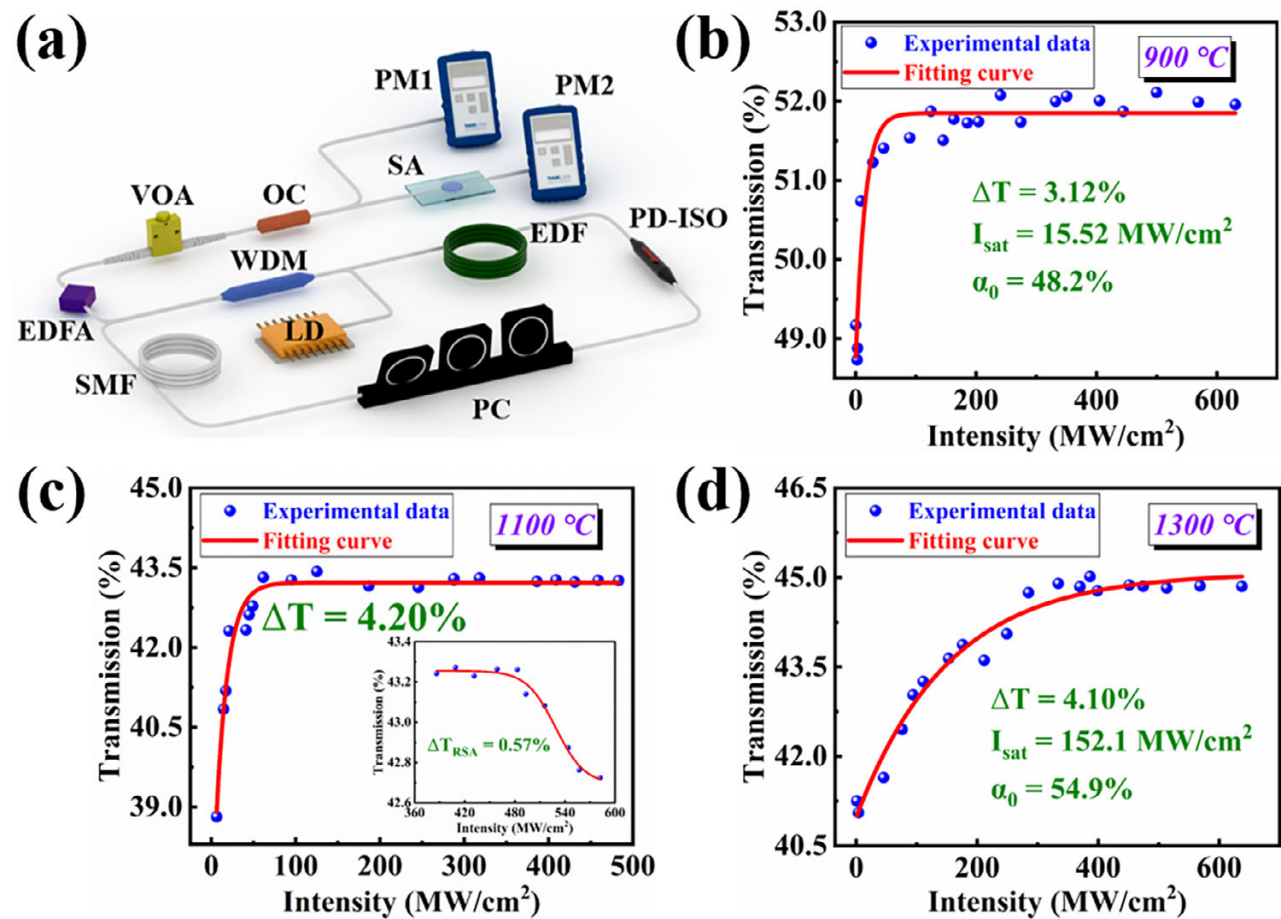
a) The schematic diagram of the in‐line balanced twin‐detector system; b) Nonlinear transmission curve of α‐Fe_2_O_3_ material calcined at 900 °C; c) Nonlinear transmission curve of Fe_2_O_3_‐H material; d) Nonlinear transmission curve of α‐Fe_2_O_3_ material calcined at 1300 °C.

According to the comprehensive characterization analysis, the Fe_2_O_3_‐H material exhibits stronger absorption in the near‐infrared band when compared to the conventional α‐Fe_2_O_3_ materials without phase transition. Notably, among the α‐Fe_2_O_3_ materials subjected to ultra‐high temperatures, the Fe_2_O_3_‐H variant demonstrates the superior crystallinity during the phase transition. To explore whether the phase transition of materials has any influence on the nonlinear optical properties of α‐Fe_2_O_3_ materials, we selected three types of α‐Fe_2_O_3_ obtained by calcination at 900, 1100, and 1300 °C to measure the nonlinear optical properties, and the results are depicted in **Figure** **3**. In Figure 3a, we constructed an in‐line balanced twin‐detector system, and the specific parameters of the system and the SAs used in this system are provided in the “Experimental Section.” In Figure 3b–d, it can be observed that when the excitation intensity employed is low, the light absorption of the α‐Fe_2_O_3_ materials gradually reduces as the excitation intensity increases. At higher excitation intensity, the absorption tends to be saturable. The saturable absorption curve can be fitted with the following Equation (1):^[^ ^57^ ^]^

(1) $$T=1-\Delta T\times \exp\left(\frac{-I}{I_{sat}}\right)-\alpha_{0}$$

where *T* is the light transmittance, *ΔT* is the modulation depth, *I* is the excitation intensity, *I_sat_* is the saturation intensity, and *α_0_* is the unsaturated loss. From Figure 3b–d, it is evident that the modulation depth of Fe_2_O_3_‐H material is 4.20%. Additionally, the saturation intensity of Fe_2_O_3_‐H material is 13.94 MW cm^−2^.

To demonstrate the superior performance of Fe_2_O_3_‐H material as SA, we have included the performance comparison between Fe_2_O_3_‐H material and other materials in the Supporting Information. As can be seen from the Table S2 (Supporting Information), Fe_2_O_3_‐H material exhibits a large modulation depth and low saturation intensity compared to other materials, highlighting its significant potential for practical applications as SA. The enhancement of the nonlinearity would be ascribed to the strong quantum confinement and local field effects from dielectric confinement and saturable excitonic resonance. Therefore, under laser irradiation, in the Fe_2_O_3_ materials undergoing a phase transition, more photons are needed to reach the bleached state, leading to the nonlinear absorption coefficient and enhanced nonlinearity. It is noting that under high excitation intensity, Fe_2_O_3_‐H material exhibits reverse saturable absorption (RSA), as shown in the illustration in Figure 3c. When the excitation intensity surpasses 400 MW cm^−2^, the transmittance of Fe_2_O_3_‐H SA decreases by 0.57%. We attribute the RSA effect may be caused by the two‐photon absorption (TPA) effect,^[^ ^58^, ^59^ ^]^ which contributes favorably to achieving high‐energy dissipative soliton resonance mode‐locking operation. However, with the calcination temperature increasing, the lattice structure deteriorates and crystallization quality diminishes, leading to structural defects that may reduce the nonlinearity of these Fe_2_O_3_ materials, as shown in Figure 3d.

#### 2.3.2 Open‐Aperture Z‐Scan System

From the aforementioned I‐scan experimental results, we established that the Fe_2_O_3_‐H material exhibits superior nonlinear optical performance as SA at 1.5 µm. To further investigate the nonlinear performance of Fe_2_O_3_‐H material comprehensively, we employed an open‐aperture Z‐scan system to measure the effective nonlinear absorption coefficient (β_eff_) of these α‐Fe_2_O_3_ materials, as illustrated in Figure S4 (Supporting Information). Figure S4a (Supporting Information) shows the experimental setup of the open‐aperture Z‐scan system used. The nonlinear polarization rotation mode‐locking fiber laser, which is identical to the in‐line balanced twin‐detector system, serves as the laser source of the open‐aperture Z‐scan system. After the pulse laser is amplified by an Er‐doped fiber amplifier, it traverses a focusing lens with a focal length of 50 mm and a quartz plate coated with prepared Fe_2_O_3_‐H material and is eventually received by the detector. Throughout the experiment, various power values can be attained by continuously adjusting the position of the quartz plate on the slide rail. The corresponding Z‐scan curve can be acquired by fitting the experimental data. The Z‐scan curve can be fitted with the following Equation (2):^[^ ^60^ ^]^

(2) $$T\;=\sum_{m=0}^{\infty }\frac{{\left[-q_{0}\left(z,0\right)\right]}^{m}}{{\left(m+1\right)}^{1.5}}\left(m\in N\right);\;\;q_{0}\;\left(z,0\right)=\frac{\beta_{eff}\cdot L_{eff}\cdot I_{0}}{1+z^{2}/z_{0}^{2}}\;$$

where *T* is the normalized transmittance, *z* is the relative distance of the sample on the *z*‐axis with respect to the focal point of the lens, *L_eff_* is the effective length of the materials, *I_0_* is the peak laser intensity, and *z_0_* is the Rayleigh length. In our open‐aperture Z‐scan experiment, the beam spot diameter is ≈100 µm, the peak laser intensity is ≈203.7 GW cm^−2^, and the effective length of the Fe_2_O_3_ materials is ≈40 µm. As can be seen from Figure S4b–d (Supporting Information), the *β*_eff_ of the Fe_2_O_3_‐H material is −0.628 cm GW^−1^. Compared with other Fe_2_O_3_ materials, it has a larger absolute value, indicating that it has a better nonlinear absorption effect, further demonstrating the more excellent nonlinear optical properties of the Fe_2_O_3_‐H material at 1.5 µm.

### 2.4 Ultrafast Mode‐Locking Operations

To investigate the application of the fabricated Fe_2_O_3_‐H material in the domain of nonlinear optics, we established a typical Er‐doped fiber resonator, as depicted in Figure S5 (Supporting Information). It employs a laser diode (LD) with an operational wavelength of 976 nm and a peak power of 450 mW as the pump source. The wavelength division multiplexer (WDM) operating at 980/1550 nm is accountable for coupling the pump light and the signal light into the same fiber. A polarization‐independent isolator (PI‐ISO) guarantees the one‐way transmission of the laser within the cavity. The polarization controller (PC) is utilized to regulate the polarization state. An optical coupler (OC) with a 10% tap ratio at 1550 nm outputs 10% of the signal light and enables the remaining light to continue oscillating within the cavity. The 1.2‐m‐long Er‐doped fiber (EDF, Fibercore I‐25) serves as the gain fiber of the resonator, and its group velocity dispersion (GVD) is 40 ps^2^ km^−1^. Additionally, the devices within the cavity are connected by the single‐mode fiber (SMF, SMF‐28e), with a GVD of −22.3 ps^2^ km^−1^ and a total length of 29.4 m. Consequently, the total length of the utilized ring resonator is 30.6 m, and a total abnormal dispersion within the cavity is −0.608 ps^2^. It is notable that when there is no SA in the resonator, the ultra‐short pulse output cannot be achieved regardless of how the output power of the pump source or the polarization state in the cavity is adjusted. This indicates that the key to achieving ultra‐short pulse output resides in the self‐made SA.

#### 2.4.1 Conventional Soliton Mode‐Locking Operation

**Figure 4:**
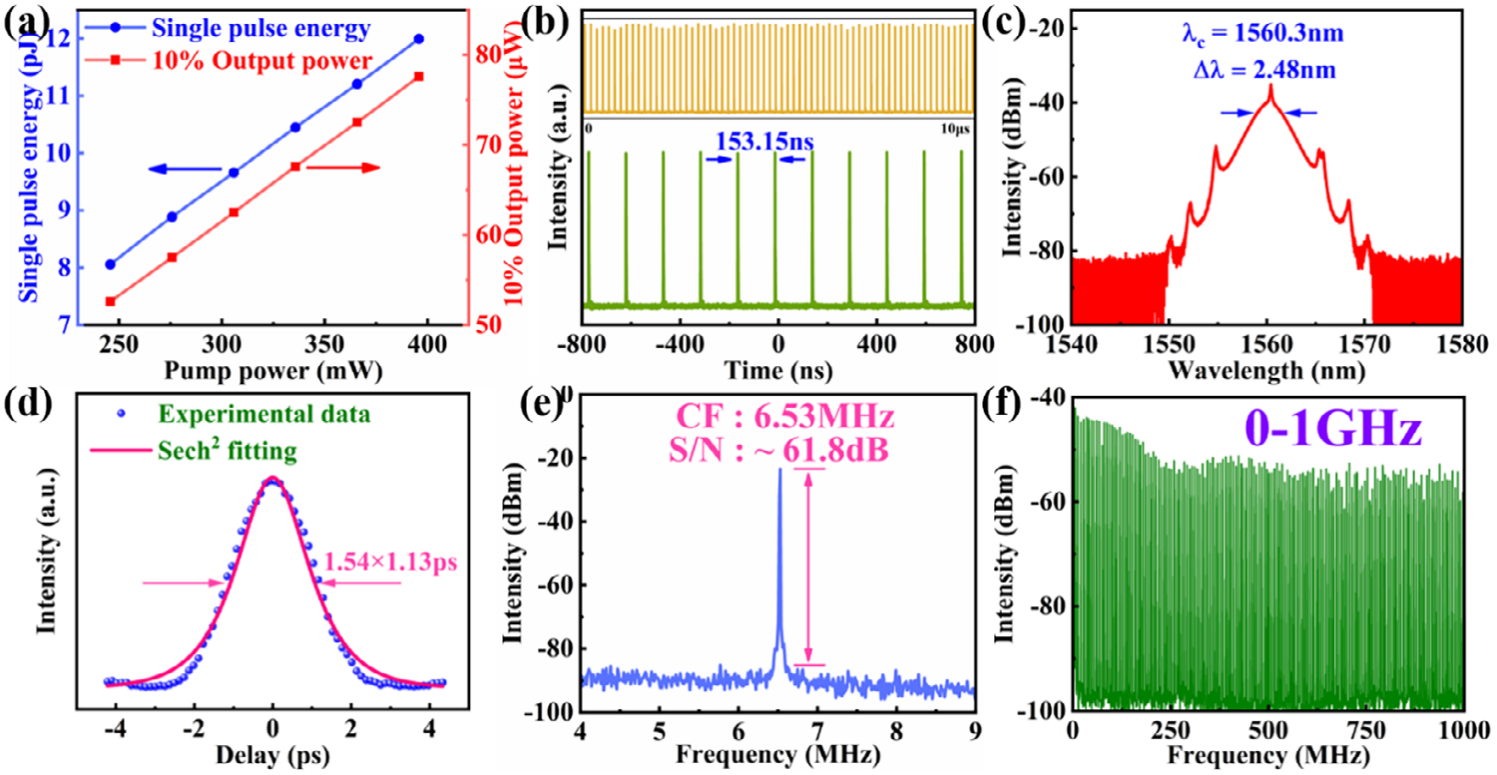
Conventional soliton mode‐locking operation of Fe_2_O_3_‐H material. a) The output power and single pulse energy vary with pump power; b) Pulse train; c) Optical spectrum; d) Autocorrelation trace; e) RF spectrum; f) RF spectrum within a span of 0–1 GHz.

Initially, we incorporate the tapered fiber without the Fe_2_O_3_ SA into the laser resonator. No mode‐locking operation is observed. Then, we incorporate the SA into the laser cavity. By gradually ramping up the pump power and meticulously adjusting the PC's state, the self‐started conventional soliton mode‐locking operation is achieved at a pump power of 245.8 mW, as depicted in **Figure** **4**. In this case, the light with high intensities can pass the Fe_2_O_3_ SA, while the low‐intensity light is absorbed, leading to the mode‐locking operation. Figure 4a illustrates the linear correlation between output power and single pulse energy with varying pump power. The increase in pump power from 245.8 to 395.8 mW results in an escalation of output power from 52.6 to 77.6 µW, accompanied by a corresponding rise in single pulse energy from 8.06 to 11.99 pJ. Moreover, Figure 4b showcases a typical pulse train spanning over 1.6 µs with a pulse interval of 153.15 ns, alongside a stable pulse train covering a time span of 10 µs, indicating the stability of mode‐locking operation.

Furthermore, Figure 4c presents the spectrum of conventional soliton mode‐locking operation featuring the central wavelength of 1560.3 nm and the 3 dB spectral width of 2.48 nm. The mode‐locking spectrum is centered at 1560 nm instead of 1530 nm, which can be attributed to the large absorption at 1530 nm, leading to the small gain. Thus the mode‐locking operation at 1560 nm is easier. It also shows the clear Kelly sidebands, which is a typical feature of lasers operating under conventional soliton mode‐locking operation.^[^ ^61^ ^]^ The enhanced resonance between the dispersive wave and the soliton wave can lead to the Kelly sidebands. The Kelly sidebands are distributed symmetrically around the spectral center peak, and their space is decided by the total dispersion value of the fiber cavity. In addition, the presence of continuous light is observed at the top of the spectrum, which comes from the interaction between the birefringent effect of the fiber and the nonlinear optical properties of SA.^[^ ^62^, ^63^ ^]^ The autocorrelation trace displayed in Figure 4d reveals that the full width at half‐maximum (FWHM) measures at ≈1.74 ps while hyperbolic secant fitting yields an estimated pulse duration of ≈1.13 ps, consequently resulting in the calculated time‐bandwidth product (TBP) slightly exceeding standard values at ≈0.345. This indicates a minor chirping effect within the pulses. Additionally, as shown in Figure 4e, we can see the radio frequency (RF) spectrum with a signal‐to‐noise ratio (SNR) of ≈61.8 dB, which has a center frequency of ≈6.53 MHz, corresponding to the pulse interval of 153.15 ns. Finally, Figure 4f provides the RF spectrum in the 1‐GHz range, further substantiating the excellent stability exhibited during conventional soliton mode‐locking operation.

#### 2.4.2 Dissipative Soliton Resonance Mode‐Locking Operation

**Figure 5:**
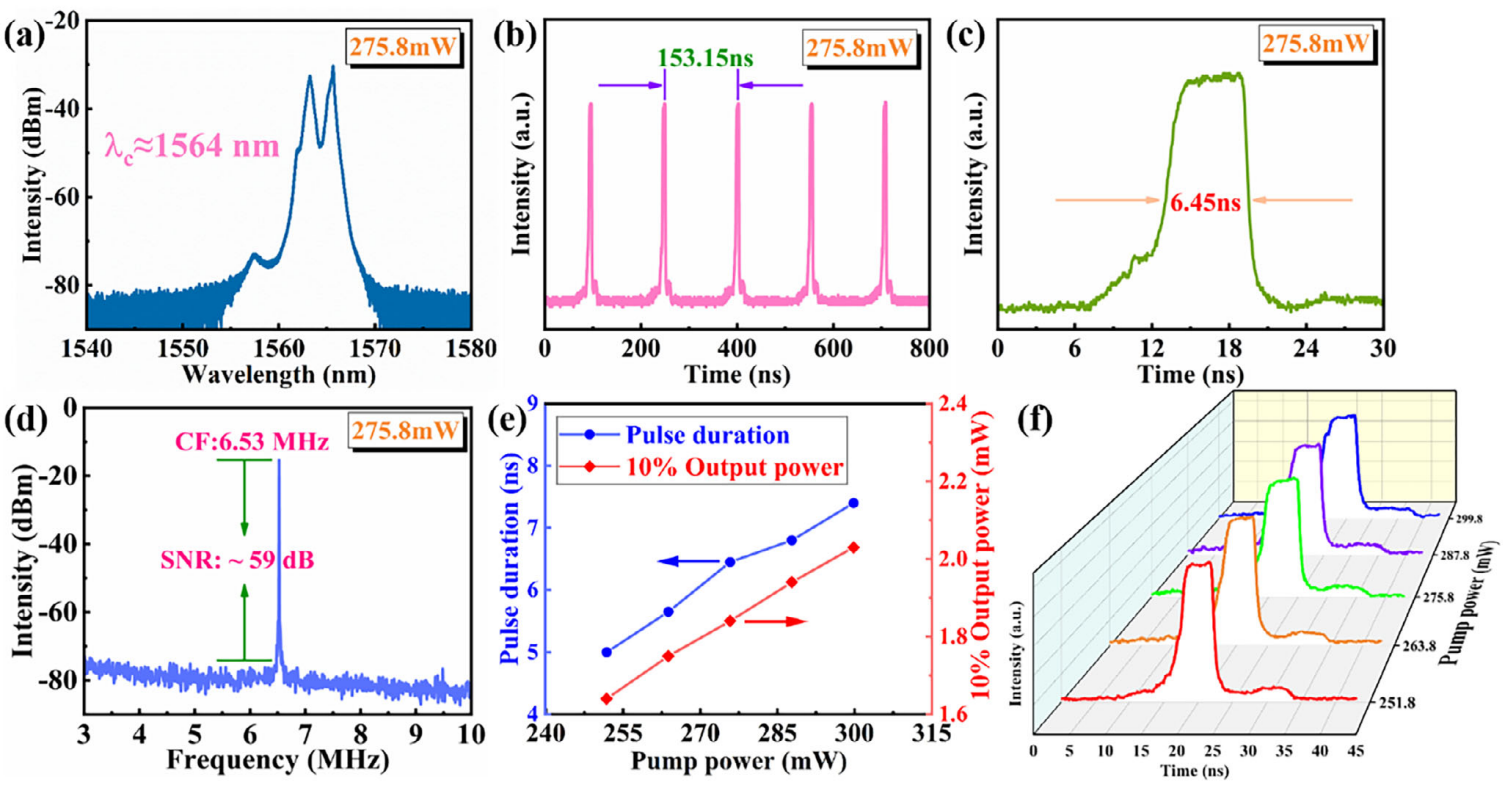
Dissipative soliton resonance mode‐locking operation of Fe_2_O_3_‐H material. a) Optical spectrum; b) Pulse train; c) Single pulse pattern; d) RF spectrum; e) The output power and pulse duration vary with pump power; f) Single pulse shape varies with pump power.

In order to further explore the potential for mode‐locking of Fe_2_O_3_‐H material in the 1.5 µm band, the dissipative soliton resonance (DSR) mode‐locking operation was finally achieved through continuous adjustment of both the pump power and polarization state, as illustrated in **Figure** **5**. Figure 5a–d presents the various mode‐locking results obtained at a pump power of 275.8 mW: Figure 5a displays the spectrum of DSR mode‐locking operation with a central wavelength ≈1564 nm. Figure 5b exhibits a typical pulse sequence spanning 800 ns with a pulse interval of 153.15 ns. The regular pulse sequence indicates the stability of the mode‐locking state. Figure 5c shows an individual single pulse over a time span of 30 ns, featuring a pulse width of 6.45 ns. As depicted in Figure 5d, the RF spectrum is shown with an SNR approximately reaching 59 dB and a center frequency measuring at 6.53 MHz. Additionally, Figure 5e depicts the output power and pulse width as a function of pump power. As the pump power increases from 251.8 to 299.8 mW, the output power rises from 1.64 to 2.03 mW and the pulse width rises from 5.0 to 7.4 ns. Among them, the direct visualization of the variation of pulse width with pump power is depicted in Figure 5f. With the continuous broadening of the DSR mode‐locking pulse, the peak power was finally clamped at a low level of ≈42 mW, and the pulse energy can be continuously increased, which is consistent with the characteristics of the DSR mode‐locking operation.

## 3 Conclusion

In this paper, a series of crystalline α‐Fe_2_O_3_ materials with different microstructures were fabricated by the high‐temperature calcination method. We discovered that α‐Fe_2_O_3_ material undergoes the phase transition at ultra‐high temperatures, leading to the formation of Fe_3_O_4_. The phase transition resulted in their optical absorption being enhanced as indicated by the characterization results. Furthermore, the results of the nonlinear optical properties tests indicated an enhancement in the modulation depth and nonlinear absorption coefficient of α‐Fe_2_O_3_ materials that had undergone phase transition. This suggested that phase transition positively influenced material modification. Notably, Fe_2_O_3_‐H material exhibited superior performance compared to others. The nonlinear absorption coefficient of Fe_2_O_3_‐H material was measured as −0.628 cm GW^−1^ using a home‐built Z‐scan setup. The modulation depth and saturation intensity of Fe_2_O_3_‐H SA were determined by a home‐built I‐scan setup, which was 4.20% and 13.94 MW cm^−2^, respectively. Finally, Fe_2_O_3_‐H SA was added into an Er‐doped fiber laser cavity, and the conventional soliton mode‐locking operation with a central wavelength of 1560.3 nm and a pulse duration of 1.13 ps, as well as the dissipative soliton resonance mode‐locking operation with a central wavelength near 1564.0 nm were obtained. Based on our experimental results, the Fe_2_O_3_‐H material obtained at ultra‐high temperature is regarded as a superior nonlinear optical material. Our research also lays the foundation for the future development of iron oxides in advanced ultrafast photonic applications.

## 4 Experimental Section

### DFT Simulation

The first‐principle simulation was performed on a VASP with PAW and PBE approximation. The self‐consistent convergence energy was lower than 1 × 10^−6^ eV, and the force convergence was within −1.5 × 10^−2^ eV A^−1^. The cutoff energy of the plane wave was 550 eV. The accuracy of the Brillouin zone sampling (K‐Mesh) was 7 × 7 × 7 with the Gamma, ensuring smooth computations. The temperature parameter is set to 1373 K.

### Preparation of α‐Fe_2_O_3_ Materials by High‐Temperature Calcination Method

A series of crystalline α‐Fe_2_O_3_ materials with different calcination temperatures were prepared by the high‐temperature calcination method. The process is as follows: Initially, a specific quantity of FeOOH powder (Merck Inc.) is placed in a corundum crucible and then positioned inside a muffle furnace. The temperature within the Muffle furnace was gradually increased at a heating rate of 200 °C h^−1^, and maintained at different final temperatures for 3 h under the air atmosphere. Subsequently, the temperature in the Muffle furnace was allowed to naturally cool to room temperature, it could get different α‐Fe_2_O_3_ materials in the crucible. The α‐Fe_2_O_3_ material with a calcination temperature of 1100 °C was named Fe_2_O_3_‐H.

### The Specific Parameters of In‐Line Balanced Twin‐Detector System

A self‐made nonlinear polarization rotation (NPR) Er‐doped fiber laser (EDFL) was used as the laser source with a central wavelength of 1534.0 nm, a repetition frequency of 11.17 MHz, and a pulse duration of 904.7 fs. The NPR mode locking fiber laser consisted of a 976 nm laser diode, a three‐in‐one device containing a wavelength division multiplexer, an optical coupler and isolator, a polarization controller, a polarization‐dependent isolator (PD‐ISO) that ensures the laser operates in the NPR mode locking state, an Er‐doped fiber and a single mode fiber. In order to ensure sufficient input power when measuring the nonlinear saturable absorption of the material, a self‐made Er‐doped fiber amplifier (EDFA) is used to further improve the intensity of the excited laser. After the excitation laser is amplified, the input excitation laser intensity can be adjusted by a variable optical attenuator (VOA). The optical coupler with a spectral ratio of 1:1 divides the excitation laser into two equal intensity sub‐beams, one of which is measured directly by the power meter (PM) as a reference light, and the other is measured by the PM as a signal light after passing through the SA.

### Fabrication of α‐Fe_2_O_3_ SAs

To prepare the saturable absorbers based on different α‐Fe_2_O_3_ materials, the different α‐Fe_2_O_3_ materials were initially thoroughly ground. Subsequently, the different ground α‐Fe_2_O_3_ powders were separately added to anhydrous ethanol solution. Then, the mixtures were subjected to ultrasonic treatment, and centrifugation was performed after the completion of the ultrasonic process. The supernatants after centrifugation were collected and reserved. Finally, the material within the supernatants was transferred to the tapered region of the homemade tapered fiber through the optical deposition method, and the requisite α‐Fe_2_O_3_ SAs could thus be obtained. Here, the tapered fiber was used has a minimum waist diameter of 12.7 µm, a tapered region length of 8.4 mm, and an original insertion loss of 46.0%.

### Characterization

The microstructure and morphology of Fe_2_O_3_‐H material were characterized by scanning electron microscopy (TESCAN MIRA LMS, Czech Republic) and transmission electron microscopy (JEOL JEM‐F200, Japan). The absorption spectrum of the materials in the ultraviolet to near‐infrared band was studied by UV–vis–NIR spectrophotometer (Shimadzu UV‐3600, Japan). The material composition and valence state of the Fe element were analyzed by Mossbauer spectrometer (ms500). The X‐ray diffraction pattern was provided by the X‐ray diffractometer (Rigaku MiniFlex600). The X‐ray photoelectron spectra of the material were obtained by X‐ray photoelectron spectroscopy (Thermo Scientific K‐Alpha).

## Conflict of Interest

The authors declare no conflict of interest.

## Author Contributions

C.H. and Z.Q. designed and performed the experiments. P.H. and P.Z. performed the formal analysis. T.Q. and Z.S. helped analyze the data. C.H. and L.D. conceived the idea and co‐supervised the project. All authors contributed to the general discussion.

## Supporting information



Supporting Information

## Data Availability

The data that support the findings of this study are available on request from the corresponding author. The data are not publicly available due to privacy or ethical restrictions.
