# Supplementary material for: Ultra‐High Temperature Calcination of Crystalline α‐Fe2O3 and Its Nonlinear Optical Properties for Ultrafast Photonics
Source: Adv Sci (Weinh). 2025 Mar 17;12(18):2500896. doi: 10.1002/advs.202500896 (PMC12079331; doi:10.1002/advs.202500896)
Supplement: Supplementary file 1 — Supporting Information [file ADVS-12-2500896-s001.docx]

**Supporting Information**

**Ultra-high temperature calcination of crystalline α-Fe_2_O_3_ and its nonlinear optical properties for ultrafast photonics**

Qingxi Zhao, Qingling Tang, Hongwei Chu*, Zhongben Pan, Han Pan, Shengzhi Zhao, and Dechun Li*

Q. Zhao, Q. Tang, H. Chu, Z. Pan, H. Pan, S. Zhao, D. Li

School of Information Science and Engineering

and Key Laboratory of Laser and Infrared System of Ministry of Education

Shandong University

Qingdao 266237, China

E-mail: [hongwei.chu@sdu.edu.cn](mailto:hongwei.chu@sdu.edu.cn); [dechun@sdu.edu.cn](mailto:dechun@sdu.edu.cn).


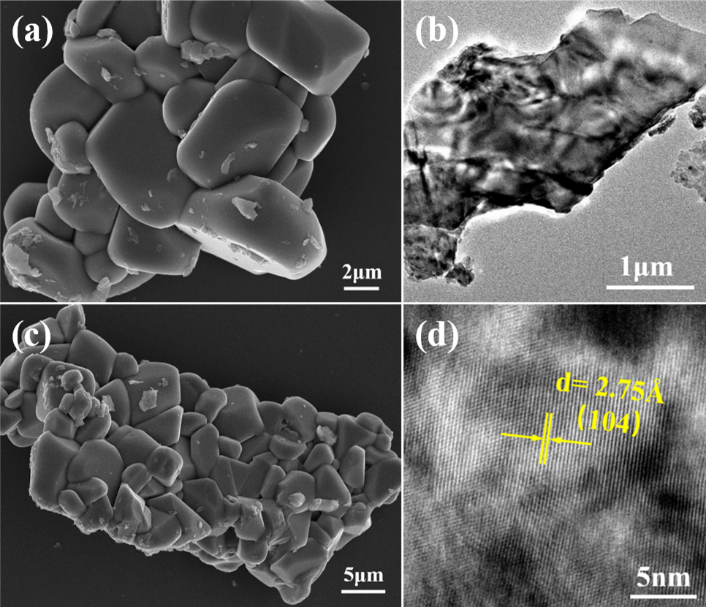


**Figure S1.** SEM images and TEM images of Fe_2_O_3_-H material.


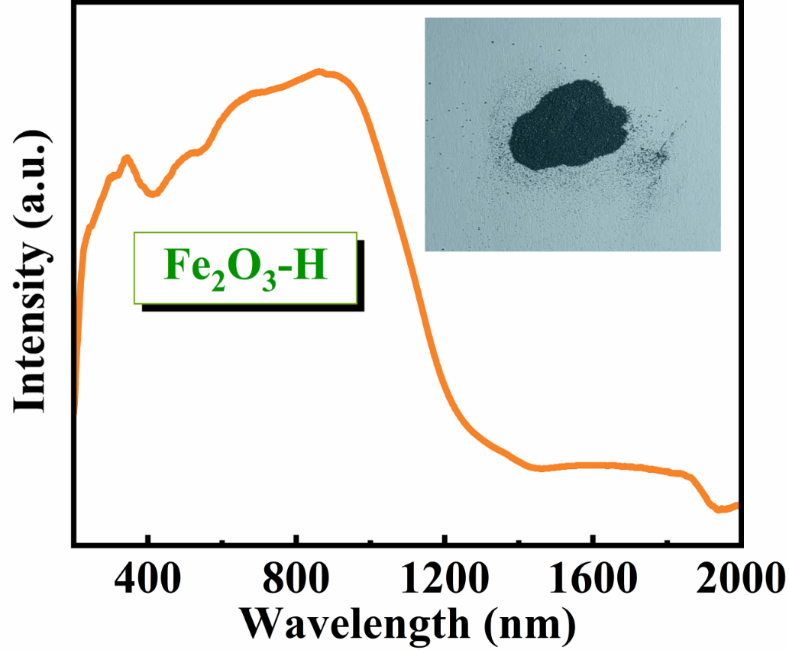


**Figure S2.** UV–vis–NIR absorption spectrum of Fe_2_O_3_-H material. The illustration is the macroscopic diagram of Fe_2_O_3_-H material.





**Figure S3.** XRD pattern of Fe_2_O_3_-H material.


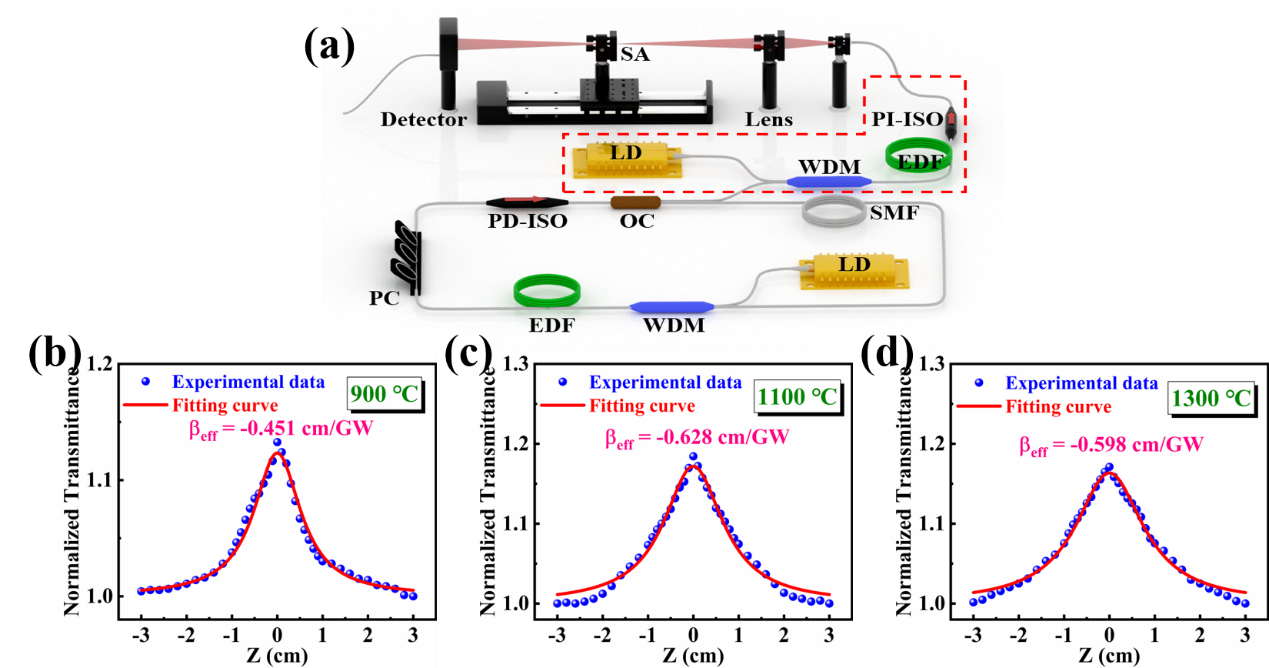


**Figure S4.** a) The experimental setup of the open-aperture Z-scan system; b) Z-scan curve of α-Fe_2_O_3_ material calcined at 900 ℃; c) Z-scan curve of Fe_2_O_3_-H material; d) Z-scan curve of α-Fe_2_O_3_ material calcined at 1300 ℃.


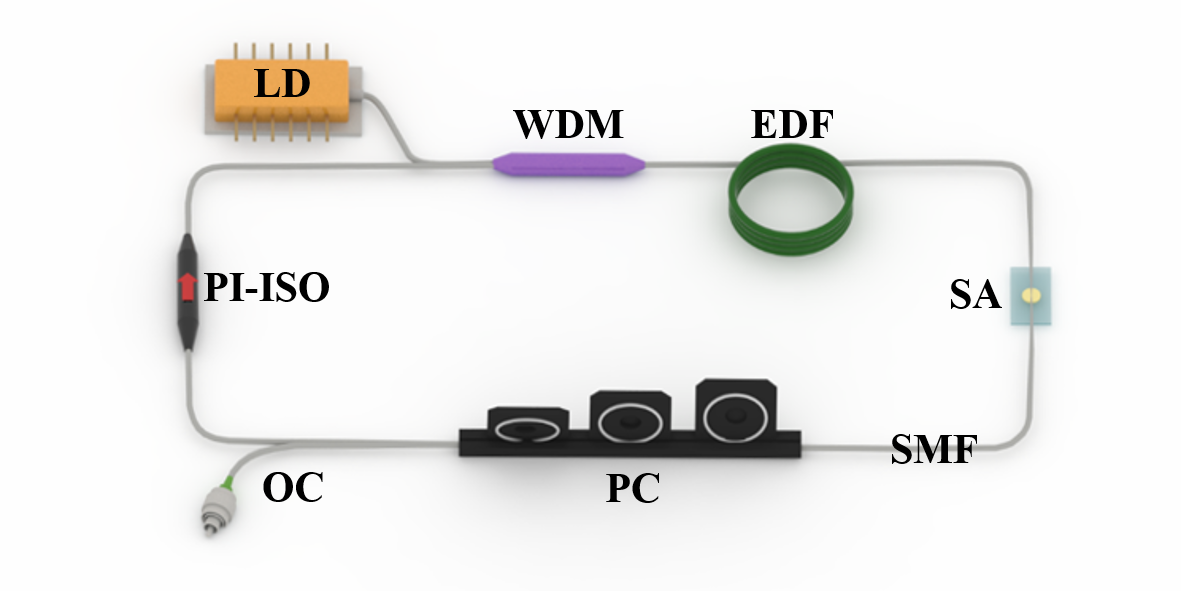


**Figure S5.** The schematic diagram of the Er-doped fiber resonator.

**Table S1. XPS fitting results of calcined α-Fe_2_O_3_ materials at different calcination temperatures.**

|  | 500 ℃ | 800 ℃ | 900 ℃ | 1000 ℃ | 1100 ℃ | 1300 ℃ | 1500 ℃ |
| --- | --- | --- | --- | --- | --- | --- | --- |
| Fe^3+^ | 36.4% | 37.7% | 38.9% | 38.7% | 38.2% | 37.2% | 36.9% |
| Fe^2+^ | 63.6% | 62.3% | 61.1% | 61.3% | 61.8% | 62.8% | 63.1% |
| O_L_ | 39% | 39% | 39% | 40% | 41% | 43% | 46% |
| O_V_ | 57% | 56% | 55% | 55% | 55% | 55% | 52% |
| O_C_ | 4% | 5% | 6% | 5% | 4% | 2% | 2% |

**Table S2. The nonlinear optical performance of SAs based on different materials in the 1.5-μm band.**

| **SA** | **Modulation Depth**  **(%)** | **Saturation Intensity**  **(MW cm^-2^)** | | **Refs** |
| --- | --- | --- | --- | --- |
| MnO_2_ | 4.40 | 32.80 | [1] | |
| Bi_2_Se_3_ | 3.90 | 12.00 | [2] | |
| SnS_2_ | 2.20 | 29.50 | [3] | |
| CoS_2_ | 3.70 | 289.30 | [4] | |
| VP | 1.57 | 68.10 | [5] | |
| β-In_2_S_3_ | 2.32 | 18.40 | [6] | |
| MXene | 0.96 | 256.90 | [7] | |
| Fe_2_O_3_ | 4.20 | 13.94 | This work | |

**References:**

[1] Y. Han, X. Li, E. Chen, M. An, Z. Song, X. Huang, X. Liu, Y. Wang, W. Zhao, *Adv. Opt. Mater.* **2022**, 10, 2201034.

[2] H. Liu, X. Zheng, M. Liu, N. Zhao, A. Luo, Z. Luo, W. Xu, H. Zhang, C. Zhao, S. Wen, *Opt. Express* **2014**, 22, 6868.

[3] T. Feng, D. Zhang, X. Li, Q. Abdul, Z. Shi, J. Lu, P. Guo, Y. Zhang, *ACS Appl. Nano Mater.* **2020**, 3, 674.

[4] L. Jing, X. Li, C. Wang, X. Wang, W. Luo, M. Qu, Y. Ren, Z. Wu, Z. Zhang, H. Chen, *ACS Appl. Nano Mater.* **2020**, 3, 9663.

[5] H. Pan, X. Ma, H. Chu, Y. Li, Z. Pan, S. Zhao, J. Zhang, D. Li, *ACS Appl. Nano Mater.* **2023**, 6, 4726.

[6] X. Li, Y. Han, Z. Shi, M. An, E. Chen, J. Feng, Q. Wang, *ACS Appl. Nano Mater.* **2022**, 5, 3229.

[7] J. Feng, X. Li, T. Feng, Y. Wang, J. Liu, H. Zhang, *Ann. Phys.* **2020**, 532, 1900437.
